# Social Drivers and Algorithmic Mechanisms on Digital Media

**DOI:** 10.1177/17456916231185057

**Published:** 2023-07-19

**Authors:** Hannah Metzler, David Garcia

**Affiliations:** 1 Center for Medical Data Science, Medical University of Vienna; 2 Complexity Science Hub Vienna, Austria; 3 Institute for Globally Distributed Open Research and Education, Vienna, Austria; 4 Department of Politics and Public Administration, University of Konstanz

**Keywords:** algorithms, digital media, well-being, polarization

## Abstract

On digital media, algorithms that process data and recommend content have become ubiquitous. Their fast and barely regulated adoption has raised concerns about their role in well-being both at the individual and collective levels. Algorithmic mechanisms on digital media are powered by social drivers, creating a feedback loop that complicates research to disentangle the role of algorithms and already existing social phenomena. Our brief overview of the current evidence on how algorithms affect well-being, misinformation, and polarization suggests that the role of algorithms in these phenomena is far from straightforward and that substantial further empirical research is needed. Existing evidence suggests that algorithms mostly reinforce existing social drivers, a finding that stresses the importance of reflecting on algorithms in the larger societal context that encompasses individualism, populist politics, and climate change. We present concrete ideas and research questions to improve algorithms on digital platforms and to investigate their role in current problems and potential solutions. Finally, we discuss how the current shift from social media to more algorithmically curated media brings both risks and opportunities if algorithms are designed for individual and societal flourishing rather than short-term profit.

## Introduction

Algorithms on digital media platforms clearly provide value, as reflected in the wealth they generate for the companies using them. They highlight relevant posts, news, people, and groups and have become necessary to reduce information overload (Narayanan, 2023b). The central role of algorithms in several types of online interaction has raised concerns that they may fuel large psychological and societal issues, specifically mental-health issues and political polarization. First, algorithms could contribute to increasing depression, anxiety, loneliness, body dissatisfaction, and even suicides by facilitating unhealthy social comparisons, addiction, poor sleep, cyberbullying, and harassment, especially in teenagers and girls (Ritchie, 2021; Twenge, 2020; Twenge et al., 2022). Second, they may fuel hate speech, fake news, and polarization by promoting extremist and populist content or by using algorithmic filter bubbles (Bliss et al., 2020; Lewis-Kraus, 2022).

Widespread usage of digital platforms and continuous interaction with algorithms could indeed affect individual and societal well-being in important ways (Büchi, 2021). However, direct evidence supporting these conclusions remains scarce (Bail, 2021; Ferguson, 2021; Sumpter, 2018). Researchers have investigated the potential effects of digital media and its algorithms using self-reports of social-media usage and digital traces of online behavior. Yet most existing studies cannot distinguish the effects of algorithms from the general use of digital media, social behavioral patterns, or large societal changes because their traces are intermingled in these types of data (Salganik, 2019).

We aim to illustrate how algorithmic mechanisms on digital media build on societal forces and how, in combination, they influence desirable and undesirable outcomes at the individual and collective levels. We focus on algorithms that determine how data is processed and what content is presented to users on digital media, rather than the more general concept of algorithms as a set of steps to perform a task. We describe the social drivers of online interaction and how algorithms might change these dynamics. We then summarize evidence and research gaps on social, algorithmic, and societal contributions for two sample topic areas: well-being and mental health at the individual level and polarization and misinformation at the collective level. Finally, we outline open questions and research opportunities to understand whether we can improve algorithms to contribute to human flourishing, and if so, how.

## Social Drivers Underlying Individual and Group Behavior on Digital Media

Social media and its algorithms are so successful because they build on ancient human needs for connection and status (Brady et al., 2020; Meshi et al., 2015; Nadkarni & Hofmann, 2012). The twin desires to get along and to get ahead are basic human motives that were crucial for survival in our ancestral environment in social groups (Cummins, 2005; Sapolsky, 2005). Status and connection are pivotal to explaining social behavior (Abele & Wojciszke, 2013; Fiske et al., 2007; Gurtman, 2009) across many domains. Examples include face perception (Todorov et al., 2008); judgments and stereotypes (Fiske et al., 2007); relationships between individuals (Schafer & Schiller, 2018) or groups (Nadler, 2016); and cultural differences in religiosity and prosociality (Gebauer et al., 2013, 2014).

Connection and status motives also strongly shape social interaction and interaction with algorithms on digital media (Eslinger et al., 2021; Meshi et al., 2015). The need for connection motivates participation in the lives of friends, interest in peer groups, self-disclosure of one’s own experiences, and renewal of old connections, as well as the pursuit of new connections, dating partners, and groups to join. Status motives influence how we broadcast content, present ourselves, receive social feedback, and observe and evaluate what others share (Burke et al., 2020; Meshi et al., 2015). Studies show that humans are susceptible to social feedback on digital media: Likes influence how quickly people post again (Lindström et al., 2021), whether or not they consider a post successful (Carr et al., 2018), and how happy, self-assured, and popular they feel after posting status updates (Rosenthal-von der Pütten et al., 2019; Zell & Moeller, 2018).

## Algorithmic Mechanisms and Other Platform Influences

All of these social motives are also ubiquitous in offline contexts, so how do algorithms and platform features change social interaction? Algorithms constantly adapt to changes in human behavior and are updated as behavior on platforms, and societal discussion about them, evolves. Humans, in turn, strive for the attention and recognition of others to gain social status, which motivates them to reproduce the behaviors that algorithms reward. The eventually observable behavior thus results from interactive feedback loops between human behavior, algorithms, and other platform features (Narayanan, 2023b; Tsvetkova et al., 2017; Wagner et al., 2021). Algorithms are designed to optimize certain metrics, which are used to rank content in user feeds or to suggest relevant accounts. Yet these optimization metrics are usually chosen to maximize the profits of corporations and advertisers (Bak-Coleman et al., 2021; Narayanan, 2023b) rather to bring about psychological and societal benefits.

The history of the Facebook algorithm illustrates how changes in metrics can affect social behavior (Merrill & Oremus, 2021; Oremus et al., 2021; Wallaroo Media, 2022), but also how little control engineers actually have over eventual outcomes within such complex emerging feedback loops (Narayanan, 2023a). In its early days, the algorithm optimized for the number of clicks, likes, and comments and the total time spent on Facebook. As users and companies learned to game the algorithm, clickbait emerged. To counter this, Facebook started maximizing the time users spent reading or watching content in 2015, which led to more passive use, more professionally produced content, less social interaction, and less sharing of original content. Because of user complaints and decreases in interaction, Facebook adapted the algorithm to encourage more “meaningful social interactions.” It boosted posts by friends and family, boosted highly commented posts, and weighted the emotional-reaction buttons much more than likes. This became problematic, as the most heavily commented posts also made people the angriest. Strongly weighting angry reactions may have favored toxic and low-quality news content. Responding to complaints, Facebook gradually reduced the angry emoji weight from five times the weight of likes in 2018 to weight 0 in 2020.

Most current digital-media algorithms strongly optimize for engagement (Narayanan, 2023b; Nikolov et al., 2019). However, social success and quality of content are only partly correlated (Salganik et al., 2006). Optimizing for popularity even seems to lower the overall quality of content (Ciampaglia et al., 2018). Engagement metrics primarily promote content that fits immediate human social, affective, and cognitive preferences and biases rather than quality content (Menczer, 2021) or long-term goals and values (Narayanan, 2023b). For instance, users are more likely to like and share low-quality content that others have already liked (Avram et al., 2020). Popularity metrics can also be gamed with inauthentic behavior, including bots, organized trolls, and fake-account networks (Pacheco et al., 2021; Sen et al., 2018). Furthermore, the interval at which an algorithm rewards behavior influences how quickly it is repeated (Lindström et al., 2021). Especially variable and unpredictable rewards, such as those on platforms with strong virality, seem more addictive (Munger, 2020b).

Other relevant platform features beyond algorithms include the vastly enlarged scale of digital compared with offline social networks. This increases audience size and magnifies differences in the influence and social status of individual users (Bak-Coleman et al., 2021). It also creates unprecedented opportunities for building connections, earning recognition, and observing others, thereby supercharging motives of social status and connection (Bail, 2021; Bak-Coleman et al., 2021; Brady et al., 2020). This increases potentially available social feedback, which notifications, likes, shares, and comments make easily accessible, immediate, and quantifiable (Brady et al., 2020). Finally, algorithm recommendations may have changed the structure of networks, increasing the frequency of triangles (Salganik, 2019; Ugander et al., 2011) and enabling interaction between distant individuals (Bak-Coleman et al., 2021).

These conditions make status comparisons particularly likely and painful (Brady et al., 2020; Munger, 2017). In large online networks, personal information about individuals is limited, whereas information about social groups is still visible. Social groups thus become the main relationships in the network, making social identities highly salient (Brady et al., 2020). Finally, larger networks mean that one encounters a larger number and diversity of individuals and opinions than in real life (Gentzkow & Shapiro, 2011; Guess et al., 2018). Digital media thus allow people to observe many (potentially very different) others and offer people unprecedented freedom to present themselves, get feedback, and adapt; they have become a central tool people use to understand themselves, understand others, and understand which groups they themselves belong to (Bail, 2021; Brady et al., 2020). As contexts in which status and groups are highly salient, digital media have become places where different groups compete for status and in-group and out-group dynamics crucially determine behavior.

When audiences are larger and more public than private, competition between groups becomes particularly strong, as discussions between political groups show, for example, on Twitter. Similarly, YouTube’s reputation for toxic comments could be linked to the extremely broad demographics of its users, leading to more conflict, and to the algorithm weighting up-votes and down-votes equally (Munn, 2020). On Facebook and especially Instagram, self-presentation is more central than group competition (Cingel et al., 2022; Midgley, 2019; Storr, 2018), leading, for example, to microcelebrities (Marwick, 2015). The TikTok algorithm guarantees a small number of views for everybody, which lowers the barriers to entry compared with the more hierarchical social networks on social media. It further makes it hard to predict which TikTok videos will go viral, which could explain long unwanted scrolling experiences, more passive watching, and less social interaction overall (Munger, 2020b).

## Social Drivers and Algorithmic Mechanisms Influencing Individual Well-Being

Mainstream discourse and parts of the scientific literature often fail to distinguish between social drivers, algorithmic mechanisms, and societal context because they fail to derive causal insights from correlation, present results limited to single studies and countries, take self-reports at face value, or omit the fact that effect sizes are small (see Cavanagh, 2017; Dienlin & Johannes, 2020; Orben & Przybylski, 2019b; Ritchie, 2021; Sumpter, 2018).

Concerns are often raised about algorithms on digital media harming mental health by fueling addiction, bad sleep, and social comparison (Smyth & Murphy, 2023), or about algorithms purposefully manipulating user mood (Booth, 2014). This debate usually conflates the time spent using social media with algorithmic effects. Only one study pinpointed algorithmic effects, finding that reducing positive posts in the Facebook feed reduces the likelihood of users posting positive content by 0.1% (Kramer et al., 2014). Indirect hints that social dynamics in online media may be more harmful to mental health than algorithms come from a natural experiment—the rollout of Facebook across U.S. colleges in 2004 to 2006 (Braghieri et al., 2022). At this time, recommender algorithms still played no role on Facebook. Yet the study observed that starting to use Facebook produced a moderate effect on depression and a small effect on anxiety disorders but no significant effect on eating disorders, suicidal thoughts, or attempts. Further results hinted that the negative effects arose from unhealthy social comparisons.

Other studies on short- or long-term well-being and mental health addressed only algorithmic effects as part of social-media usage as a whole. Two randomized controlled trials testing the effects of deactivating Facebook (Allcott et al., 2020; Asimovic et al., 2021) observed small to moderate decreases in anxiety, one in depression, and one in loneliness. Many other emotions did not change, consistent with an experience-sampling study testing the effects of using Twitter (de Mello et al., 2022). Life satisfaction did not change after deactivating Facebook for 1 week (Asimovic et al., 2021), but increased after 4 weeks (Allcott et al., 2020). Furthermore, specification curve analyses showed very small negative associations with social-media usage in adolescents (Orben et al., 2019; Orben & Przybylski, 2019a).

Overall, the debate about social media and individual well-being requires more nuance. Evidence for algorithms driving or reinforcing unhealthy dynamics is very thin, supporting, at best, a small effect on mood. Instead, unfavorable social-status comparisons online may harm mental health. The direction of effects between social-media usage and mental health is unclear (Ferguson, 2021; Luhmann et al., 2022; Orben et al., 2019); the direction and size of effects depend on who uses social media and in what way (Büchi, 2021). For instance, teenage girls or already socially disadvantaged individuals may be particularly vulnerable (Allcott et al., 2020; Heffer et al., 2019; Midgley, 2019; Orben et al., 2019, 2022). Passive, extreme, or low use is related to poorer well-being, whereas active, social, and moderate use correlates with better well-being (Dienlin & Johannes, 2020). Furthermore, self-reports of usage and addiction do not reliably measure actual usage and tend to systematically overestimate them, more so in some users (such as girls) than others (Boyle et al., 2022; Mahalingham et al., 2022; Scharkow, 2016; Shaw et al., 2020).

Yet algorithmic effects could also emerge as slow trends or at higher levels of the complex system involving digital media and the offline world; studies on individuals in limited time periods cannot capture such effects. For example, the constant opportunity to express oneself could slowly affect independent emotion-regulation abilities, and algorithmic reinforcement of emotional content could change norms of emotional self-disclosure in relationships over time. In any case, potential risks to the well-being and mental health of vulnerable groups need to be taken seriously, and large corporations should be held responsible for preventing harm.

None of the cited studies says anything about the societal context, including achievement pressure and individualization increasing with neoliberalism (Levitz, 2023; Storr, 2018); increasing economic inequality and insecurity (Wilkinson & Pickett, 2017); more single households in wealthy societies, driving loneliness; sleep irregularities and addiction, especially in younger adults (Cocco, 2022); or general uncertainties about the future (e.g., climate change; Ingle & Mikulewicz, 2020). All of these societal developments could crucially affect mental health, with algorithms reinforcing existing dynamics but not being the primary cause.

When problems have strong social or societal root causes, solutions will require difficult political, institutional, and economic changes. To address the actual causes, we need research that disentangles which, if any, of the issues currently blamed on algorithms are driven by social dynamics or societal context. The influence of societal context is particularly difficult to pin down. It would require large-scale and longitudinal studies tracing and separating multiple interacting factors and their online and offline effects over time, including algorithmic and societal changes across platforms, nations, and cultures. Data for such studies are currently not available, but the European Digital Services Act may be a step forward (Turillazzi et al., 2023).

## Social Drivers and Algorithmic Mechanisms Influencing the Collective Dynamics of Political Polarization and Misinformation

Could algorithms foster echo chambers of like-minded people and polarization (Garimella et al., 2017)? Do engagement metrics promote hate speech, radicalized content, and fake news? We highlight a few studies that help dissociate algorithmic mechanisms from social drivers. For more details, see Van Bavel et al. (2021), Ferguson (2021), and Lorenz-Spreen et al. (2022).

Online echo chambers might have a more minor role than has been commonly assumed (Bakshy et al., 2015; Bruns, 2021; Guess et al., 2018; Sumpter, 2018; Törnberg, 2022) and are smaller than offline echo chambers (Gentzkow & Shapiro, 2011). Weaker online echo chambers mean that people are exposed to more people they disagree with. Similarly, digital media may increase perceived rather than actual polarization (Bail, 2021). Supporting this, a field experiment on U.S. Twitter observed increased political polarization after exposure to posts from opinion leaders of the opposing party (Bail et al., 2018) and experience sampling reveals consistent results (de Mello et al., 2022).

Increases in actual polarization are less bad than commonly assumed; there is still overlap for substantial issues in the views of political parties (Bail, 2021). Because misinformation is largely a symptom of polarization (Altay, 2022; Osmundsen et al., 2021; Petersen et al., 2022), exposure to online misinformation might also have been overestimated. Misinformation accounts for a small proportion of digital-news consumption (Altay, Nielsen, & Fletcher, 2022) and is mostly shared by a tiny minority of users (Grinberg et al., 2019; Osmundsen et al., 2021). Additionally, misinformation has been shown not to easily change beliefs or political voting behavior (Bail et al., 2020; Guess et al., 2020).

Regarding specific algorithm effects, a study on Facebook data in 2014 (Bakshy et al., 2015) found that users’ social networks determined posts in their feeds much more strongly than the algorithm. Similarly, users actively engage with more partisan news than suggested by the Google search algorithm (Robertson et al., 2023). The YouTube algorithm also does not seem to radicalize many users: Only 1 out of 100,000 who started viewing moderate content later moved to far-right content (Ribeiro et al., 2021). Most movement to far-right videos comes from outside the platform, and far-right videos are not more likely toward the end of sessions, where algorithmic recommendations matter most (Hosseinmardi et al., 2021). Instead, the demand for far-right content, with supply being easy, and the lack of more moderate conservative content may explain the increases in views of such content until mid-2017 (Munger & Phillips, 2020).

Overall, evidence neither shows that algorithms cause echo chambers, nor that echo chambers cause polarization. Yet algorithms can still contribute to polarization—for example, by weakening echo chambers and exposing people to more views they disagree with. Current evidence is consistent with the view that digital media as a whole, including algorithms, fuels perceived polarization by making extremist voices more visible and hiding moderate majorities (Bail, 2021). Two randomized controlled trials support this: In the politically polarized United States, affective polarization decreased after Facebook abstinence (Allcott et al., 2020). However, not having online contact with (probably moderate) ethnic out-group members in Bosnia-Herzegovina increased affective polarization (Asimovic et al., 2021). Similar to the idea of perceived polarization increasing actual polarization, the myth of fake news being common makes people more skeptical of news in general (Altay, Berriche, & Acerbi, 2022; Fletcher & Nielsen, 2019; Guess et al., 2021). Again, most studies on digital-media effects say little about larger societal drivers of polarization. One likely driver of increasing affective polarization, and thus misinformation, is the rise of authoritarian populism in many Western countries, which itself may arise from economic insecurities or backlash to progressive cultural change (Inglehart & Norris, 2016; but see Schäfer, 2022).

Yet such societal developments can interact with algorithmic effects by affecting discourse and decisions about algorithms. Letting platforms decide how to rank content may have seemed obvious for a long time, but discussions about this are increasing. Additionally, currently polarized or populist debates may make it difficult to find common ground on algorithmic optimization metrics, making it harder to address potentially negative effects. Similar feedback loops in the positive direction could begin with algorithms that emphasize the overlap in views of political groups, which could reduce polarization. Furthermore, algorithms that emphasize nuanced content could help decrease paralyzing climate anxieties or highlight constructive perspectives that motivate action. Finally, algorithms could create more collective emotional experiences by facilitating the spreading of emotional content. This could motivate protest movements or prosocial behavior but also foster intergroup conflict and intolerance.

## Research Avenues Toward Solutions and Flourishing

Digital-media companies benefit from the narrative of omnipotent algorithms, as their business model relies on their customers (i.e., advertisers) believing it (Munger, 2020a; Sumpter, 2018). For instance, Cambridge Analytica wanted its customers to believe they could shift political opinion in the crucial target group of undecided voters (Sumpter, 2018). Munger (2020a) argued that activists and society should stop buying this story. Silicon Valley corporations should carry responsibility for evaluating the potential societal consequences of their platforms. Still, blaming technology as the supposed mechanism behind a problem without looking at the drivers that power the problem is unlikely to lead to resolution. This approach directs attention away from actual root causes and potentially misleads societal discourse and policies, creating ground for further complaints.

Famous platform critics such as Francis Haugen or Elon Musk (Oremus et al., 2021; Riemer & Peter, 2022) have suggested getting rid of algorithms entirely and returning to reverse chronological ordering of posts. However, chronological order is just another kind of algorithm with its own drawbacks (Riemer & Peter, 2022): It favors more frequent posters, does not reduce information overload, and likely implies that users will miss more carefully prepared but rarer content. Getting rid of algorithms also means not using them as tools where they are indeed useful. Using algorithms well, in turn, requires developing shared visions and values—things users want algorithms to align with—which is a major important avenue for future research.

Future researchers need to develop and test theories about the role of algorithms (see Box 1), including potentially positive contributions and the mechanisms and outcomes of feedback loops with social behavior. Algorithms could even help to solve problems to which they currently contribute, and they can be intentionally designed to foster short- and long-term well-being and flourishing (Steinert & Dennis, 2022). This requires developing a vision for digital-media design and algorithm design beyond those proposed by existing for-profit companies (Bail, 2021; Büchi, 2021).

**Box 1.** Some Important Psychological Research Questions on Algorithms on Digital Media

**Overarching questions** • With which values and purposes do we want the outcomes of our algorithms to align? • How can psychological knowledge about social behavior and cognition help to design algorithms and platforms to best foster human well-being and flourishing at an individual and collective level? • Do current algorithms on digital media have beneficial effects compared with media without algorithms? • Which new risks and opportunities arise from the current shift from social to algorithmic media? • Which platform design and algorithm features best align with different purposes? Which purposes require different digital environments, and which can be combined? • How does giving users choices about algorithm metrics or other design features affect individual and collective well-being? Which choices should be made available, and which should be implemented broadly? How should these choices be assessed? **Individual and interindividual well-being, happiness, and flourishing** • What are specific algorithmic effects on emotions and mental health, independent from social-media usage as a whole? • How could algorithms and other platform design features . . . ○ foster short- or long-term well-being and flourishing of individuals (including pleasure, happiness, life satisfaction, and connection)? ○ emphasize connection over social comparison between individuals? ○ foster new and deepen existing important relationships? ○ reward interindividual empathy, support, and prosocial behavior? ○ foster successful emotion regulation or collective emotional experiences? • How would allowing users to choose what they want to see affect their well-being? **Social cohesion, nuanced political discussion, and high-quality information** • Do algorithms contribute to increasing affective polarization by fueling perceived polarization—for example, by suggesting more extreme political content? • How could algorithms and platforms . . . ○ make silent moderate majorities more visible? ○ reduce the visibility of extremist, toxic, outraging, or regrettable content? ○ promote nuanced and high-quality content? ○ reward expressions of empathy and understanding? ○ reward cooperation rather than status competition between groups? ○ promote constructive online intergroup contact? • How would allowing individual user or community choices on algorithmic ranking affect polarization and the spread of misinformation at the macroscale on digital platforms?

Although problem audits of algorithms are rare, studies on beneficial effects are even rarer. Some A/B tests on beneficial outcomes exist for interface design (e.g., Zhang et al., 2022), content manipulations and connection recommendations (e.g., Rajkumar et al., 2022), or for achieving collective outcomes with the help of random bots (Shirado & Christakis, 2017). However, more experiments comparing different optimization algorithms and comparing platforms with and without algorithms are needed.

Testing the effects of current and possible future algorithm and platform design requires platforms that allow experimental manipulation while obtaining users’ consent. Computational social scientists have begun developing such bespoke social-media platforms to test the effect of concealing political affiliation or gender identity (Combs, Tierney, Alqabandi, et al., 2022; Combs, Tierney, Guay, et al., 2022), social-engagement metrics (Avram et al., 2020), or anti-addictive design features (Zhang et al., 2022). Collaborations between academia and existing platforms are another promising approach (Stray & Hadfield, 2023).

### Ideas for algorithm and platform design to foster flourishing

Algorithms can improve digital-media platforms in two ways: by using different optimization metrics to rank content, or by prompting interventions upon detecting problematic content. Current digital-media platforms show that engagement metrics that optimize for entertainment are unlikely to foster rational debates. Optimal design choices will thus likely depend on the purpose of a platform and potentially on user preferences. We may want to create different platforms to foster nuanced political discussion, amplify entertainment and short-term pleasure, promote regular contact between friends and relatives, deepen personal relationships, or build communities (e.g., for mental-health support). Future research on which design choices work best to achieve each purpose, and which ones require separate platforms or subspaces on existing platforms, would be very valuable.

Platforms for fostering nuanced political discussions that strengthen social cohesion, moderate voices, and diversity will have to focus on reducing perceived polarization. This requires reducing the visibility of strongly partisan and triggering content (Rose-Stockwell, 2018), perhaps with algorithm metrics that prioritize content popular on both sides of the political spectrum. This would highlight moderate voices and reveal the opinion overlap for the important issues where it actually exists (Bail, 2021), and could promote more trustworthy news sources (Bhadani et al., 2022). Similar algorithms could highlight which principles or practical approaches resonate with people on both sides of other belief spectrums, such as those relating to climate change or alternative medicine.

Algorithm rankings could further foster intergroup contact and understanding by presenting posts that are not too distant from a user’s own position (Levendusky, 2018; Sherif, 1963). In this way, algorithms could support small steps toward understanding alternative views. Using algorithmic estimates of users’ positions on a dimension, platforms could further label extreme voices as such, give users feedback about their own position, or show how moderate and extreme users on both sides have responded to an account or post (Bail, 2021). Other suggestions include toning down status incentives by hiding or reducing the visibility of engagement metrics for certain types of posts (Avram et al., 2020) or adding cues that spotlight passive user behaviors (e.g., how many scrolled over a post; Lorenz-Spreen et al., 2020). Twitter introduced view counts for tweets early in 2023, creating research opportunities to explore how this affects social-reward experiences or the spreading of polarizing and untrustworthy content. Finally, anonymity is a promising nonalgorithmic design feature for reducing conflicts rooted in social identity, with the potential to make discussions on controversial issues kinder (Combs, Tierney, Guay, et al., 2022).

Optimizing algorithms for metrics such as civility (Lewandowsky & Kozyreva, 2022; Oremus et al., 2021) would require defining what counts as civil and how civility fosters democratic discourse and diversity. When a minority is unjustly neglected, or an elite unfairly privileged, for example, angry responses are appropriate and necessary. Rather than deciding which values should guide the choice of algorithm metrics, platforms could also let users define values themselves (Lewandowsky & Kozyreva, 2022; Lorenz-Spreen et al., 2020). Facebook has tested such an approach in its “breaking the glass” experiments, deploying an algorithm that emphasized posts that users considered to be “good for the world” (Roose et al., 2021). Although this reduced low-quality content, it also lowered how often users opened Facebook and was therefore implemented only in a weakened version.

A second way to use algorithms is to detect certain posts or activities and then trigger interventions. The simplest of all interventions is adding friction, that is, increasing the time or effort it takes to share content (Brady et al., 2020; Lorenz-Spreen et al., 2020; Menczer, 2021). Adding friction seems particularly useful to prevent impulsive sharing of sensational news and outraged or toxic comments. In some cases, simply adding a time gap before allowing users to post or share might suffice. In others, additional prompts could encourage reflection before sharing (Rose-Stockwell, 2018). Empathic and humanizing prompts have been shown to reduce affective polarization (Saveski et al., 2022) and racist harassment (Hangartner et al., 2021; Munger, 2017). Undo prompts after posting hateful comments, default options to turn comments from public to private, or ideological prompts explaining that posts with moral-emotional language are unlikely to reach the other side, could all reduce hateful content (Rose-Stockwell, 2018). Interventions that effectively reduce affective polarization provide further inspiration (Hartman et al., 2022; Voelkel et al., 2022).

To foster mental health and healthy usage of digital media, algorithms can detect linguistic markers of symptoms or certain activity patterns. Trying to detect users at risk of mental-health issues, with the goal of then providing contact points for support, is a popular research field, which, however, urgently requires methodological-validation efforts (Chancellor & De Choudhury, 2020). To reduce unwanted addictive use, algorithms can encourage users to disengage by providing reminder bots after excessive periods of scrolling or providing usage statistics (Zhang et al., 2022). Interventions such as reading-progress indicators, feed filters and content blockers for specific types of content, and separate topic-focused feeds instead of one main feed, seem even more effective (Zhang et al., 2022), and could be improved via algorithmic suggestions. Finally, we do not know of any research on how changing algorithm metrics could support individual well-being. Algorithms that reduce the visibility of toxic, regrettable, and outraged content may help reduce content that negatively affects well-being (Rose-Stockwell, 2018). Research on algorithms that prioritize content from important personal contacts, expressions of empathy and connection, or prosocial behaviors could contribute to positive well-being outcomes.

### Choosing values, validating metrics, and evaluating their effect on outcomes

As the above discussion illustrates, many different values are potentially justifiable candidates for algorithmic optimization. Choosing such values, validating the metrics to optimize for them, and testing their effect on various outcomes will require research in cultural, moral, social, political, affective, and clinical psychology, as well as computational, sociological, political, and economic approaches. Such research needs to determine for which values societal consensus is possible, and where digital media have to accommodate different needs, visions, and goals within the same or between different platforms. It should further explore which values and goals individuals prioritize and how social and cultural norms affect these processes in communities and societies.

Research could also compare different ways of assessing these value preferences: Avoiding perpetuating the influence of social and cognitive biases will probably require asking for user decisions in advance and at an abstract level, rather than measuring immediate preferences when users thoughtlessly scroll through their feeds. Preliminary research shows that most U.S. users across political and demographic groups opt for seeing more accurate, nuanced, friendly, positive, and educational content (Rathje et al., 2022), although such content currently does not typically go viral by itself. Researchers need to test whether users would actually make the choices they report preferring on the platforms they regularly use, and they then need to determine whether this would reduce misinformation and polarization at the macroscale of digital platforms. They further need to explore where individual user or community choices on algorithmic rankings or interventions are possible and beneficial (Lewandowsky & Kozyreva, 2022; Lorenz-Spreen et al., 2020), and where they need to be restricted. For example, letting users opt for only partisan content is dangerous, as contact with moderate voices from other groups may be necessary to reduce polarization.

Once certain values are agreed upon, methodological research can be employed to validate which metrics could actually represent those values, relying on the digital trace data available to algorithms. Finally, empirical research should be used to investigate how different metrics would affect the various outcomes, including affective well-being, mental health, societal cohesion, and nuanced political discussions. Given that social media are complex systems with emerging feedback loops between social drivers and algorithms, this research needs to incorporate methods from complexity science and computational social science, such as network analysis or agent-based modeling (Borsboom et al., 2021; Jackson et al., 2017; Smith & Conrey, 2007; Vlasceanu et al., 2018) to address these many open questions.

### Shift from social to algorithmic media

Twitter, Facebook, and Instagram could be referred to as *traditional social media*, as their information-distribution mechanism relies primarily on social networks (Mignano, 2022; Munger, 2020b). In contrast, other platforms like YouTube and TikTok mostly rely on recommendation algorithms instead of social links. On traditional social media, social drivers can have a much larger influence on interactions and spreading dynamics. In contrast, on *algorithmic media*, the platform itself has much more power to determine presented content through the recommender system and content feed (Mignano, 2022; Narayanan, 2023b). Algorithms are more economically competitive as information-distribution mechanisms because social graphs are now easily available (Mignano, 2022). Likely for this reason, Facebook and Instagram have started following TikTok’s example by adding short recommendation-based video feeds. This trend may entirely change our current conclusions about the algorithmic effects that have been limited so far. Algorithmic media might worsen problems like addiction or propaganda. Munger (2020b) has argued that the immersive nature of TikTok’s mobile-first design, its higher capacity to evoke emotions via both visual and audio information, the ease of posting content, and the unpredictable virality of its algorithm might make it more addictive and its users more vulnerable to political persuasion.

However, the shift from social to algorithmic media may also present an opportunity for the endeavor of designing digital media that foster human flourishing. Because algorithmic content distribution gives greater control to the platform compared with popular users, algorithms could select content on the basis of metrics that foster well-being of individuals and societies. They could highlight overlap between opposed groups (Bail, 2021), prioritize news a user actually wants to see (Rathje et al., 2022), or simply limit how far fake news spreads (Bak-Coleman et al., 2022). Both new risks and opportunities arising from algorithmic media are important avenues for future research in psychology and computational social science.

If algorithms make societally relevant decisions, it becomes pivotal who takes these decisions, and in what way. Making sure these decisions benefit society will require transparency about algorithmic design (Kozyreva et al., 2021; Wagner et al., 2021). The recent release of the code of the Twitter algorithm illustrates that in order to actually evaluate effects we need not only information about how the algorithms weigh types of content and interactions but also information about the machine-learning models that make suggestions for individual users (Narayanan, 2023a). Although we see potential in beneficially using algorithms on digital media, we must acknowledge the barriers that exist for this kind of research. Because the vast majority of online media are proprietary for-profit platforms, the designs and targets we presented are likely at odds with profit-making to a certain extent. Testing, implementing, and adopting solutions will therefore likely require regulation (Gal, 2022). Given the unique role of digital media in creating a public sphere in a globalized world, researchers and activists have even discussed whether digital media should become a public good (Fournier-Tombs, 2022). However, we are also still very early in the history of digital-media platforms, with large shifts of users to new platforms every couple of years (Bail, 2021). Over time, market dynamics could still play out in ways that better satisfy user preferences beyond short-term rewards.

## Conclusion

We have outlined different ways in which algorithms on digital media could promote positive emotions, mental health, social cohesion, and nuanced discourse. In the context of a globalized world, polarized democracies, and increasingly individualized societies, efforts to design algorithms that foster intergroup contact via digital media may make valuable contributions to reduce social, ethnic, political, and cultural barriers.

## Transparency

*Action Editor:* Melanie Mitchell

*Editor:* Interim Editorial Panel
